# Evolutionary dynamics of a four-stakeholder ecosystem for misinformation governance on short-video platforms

**DOI:** 10.1098/rsos.261361

**Published:** 2026-10-28

**Authors:** Xiaoyu Song, Wenjie Song

**Affiliations:** ^1^ School of Economics and Management, Yanshan University, Qinhuangdao, People's Republic of China; ^2^ School of Public Administration, Yanshan University, Qinhuangdao, People's Republic of China

**Keywords:** evolutionary game theory, computational modelling, misinformation governance, platform governance, socio-technical systems, governance ecosystem

## Abstract

Misinformation governance on short-video platforms depends on strategic interaction among online media, netizens, platforms and government regulators, but platform decisions are often treated as exogenous in two- and three-party models. We develop a quadripartite evolutionary game model to examine these interactions under bounded rationality. Equilibrium analysis and numerical simulations identify the stable strategy profiles and the conditions under which they emerge. Under the baseline parameters, the system has a unique asymptotically stable profile. Platforms adopt active governance, media report without verification, government regulators apply loose supervision and netizens assess information rationally. Parameter analysis shows that higher platform traffic revenue, a greater probability of insider refutation, and larger credibility and reputational losses accelerate convergence to this profile. Strict supervision can alter the transient evolutionary path and accelerate convergence under the benchmark conditions, but it is not part of the stable profile. Within the model, active platform governance and user rationality are the principal stabilizing factors, while government supervision plays a complementary role. These results provide a modelling basis for coordinating platform, user and regulatory responses to misinformation in short-video ecosystems.

## 1. Introduction

In recent years, short-video platforms have emerged as crucial arenas for digital information dissemination and the evolution of online public opinion. Supported by algorithmic recommendation, immersive communication and highly fragmented information presentation, mainstream short-video and social media platforms have profoundly reshaped how the public accesses, spreads and consumes information. Compared with traditional social media, short-video platforms feature stronger emotional appeal, faster information diffusion and higher user engagement. While these characteristics improve the efficiency of information transmission, they also greatly accelerate the spread of misinformation.

Driven by the ‘attention economy’, some self-media creators tend to attract user attention through emotional narration, exaggerated expression and the recombination of unverified information in pursuit of traffic and exposure. Given that short-video content usually carries strong visual impact, shorter transmission chains and significant algorithm-amplification effects, misinformation can spread extensively in a short period, further undermining public judgement and social trust. Especially in emergent public events and hot social issues, misinformation propagation not only disrupts the online ecosystem but may also trigger social panic and governance risks. Understanding how misinformation spreads, and how different actors can respond, is therefore central to platform governance.

Existing studies have mainly focused on the propagation mechanisms of online misinformation, platform governance models and government regulatory strategies, and widely adopted propagation dynamic models and evolutionary game theory to analyse the multi-agent decision-making process. However, most studies concentrate on bilateral or trilateral interactions among the government, media and netizens, paying insufficient attention to the core role of short-video platforms in content moderation, algorithmic recommendation and content governance. Moreover, research on the propagation characteristics of misinformation in short-video scenarios remains limited, and the dynamic synergy mechanism among platform governance, user cognition and government supervision has not been fully revealed.

This study models short-video misinformation governance as a dynamic socio-technical system. The model extends evolutionary game analysis to four actors: online media, netizens, short-video platforms and government regulators. It describes their interdependent decisions under bounded rationality. Equilibrium stability and evolutionary paths are then analysed, and numerical simulations assess how key parameters reshape those paths. The framing follows the social physics view of digital governance as a collective socio-technical process [1]. Treating the platform as a strategic actor with its own incentives allows the analysis to account for feedback between moderation, user behaviour and regulation. Because the model is specified in general terms, its conclusions can be adapted to different short-video contexts rather than tied to one platform.

## 2. Related research

Research on social media misinformation has concentrated on exposure, diffusion and platform governance. Recommendation systems can reinforce selective exposure and political polarization [2]. Pandemic-era evidence shows that social media amplified false claims [3]. Related work on echo chambers found that interaction within ideologically homogeneous communities can accelerate diffusion [4]. Detection studies provided complementary tools for identifying fake news [5]. Reviews by Zhou & Zafarani [6] and Kumar & Shah [7] mapped the broader governance challenge. As audiovisual platforms have expanded, research has turned to regulation and moderation [8–12]. Katsirea [8] highlighted regulatory concerns, while Zeng & Schäfer [9] linked recommendation architecture to ‘dark platforms’. Gillespie [10] emphasized moderation and content management, and studies of deplatforming examined whether removing harmful accounts reduces the spread of harmful content [11,12]. This work shows that platform architecture and moderation policies can shape the visibility of misinformation.

Modelling research on misinformation governance has developed along two lines. Diffusion models describe how misinformation and corrections compete over time. Jiang *et al*. [13] used ordinary differential equations, while later models incorporated conformity [14] and user preference selection [15]. Evolutionary game models shift the focus to strategic choice. A group of studies [16–18] developed frameworks for multi-agent governance. Chen & Huang [19] combined evolutionary game theory with an SEIQR model to analyse online public opinion during emergencies. Prospect-theory extensions [20–22] examined three-party interactions among government, media and netizens. Later work considers interaction costs [23] and combinations of incentives, reputation and platform regulation [24–26]. Wang *et al*. [27] integrated social media user data with an SHIR diffusion model to assess official clarification and platform governance. Related applications include environmental regulation and elderly care [28,29]. Platform-mediated environments add a further complication because dissemination, interaction, and recommendation operate across coupled network layers [30].

These studies clarify important mechanisms, but they leave the platform's strategic role underexplored. Most game models focus on two or three actors, typically government, media and netizens. Even when platforms appear, they are often treated as moderators or channels rather than actors whose decisions respond to incentives and feedback. Existing models also rarely capture the distinctive features of short-video systems. Immersive presentation, emotional contagion and recommendation amplification interact with user behaviour and regulatory pressure, yet they are not analysed together. The resulting feedback loops among media, platforms, government and netizens therefore remain unclear. This omission limits understanding of how governance outcomes emerge from their interaction.

## 3. Construction of a quadripartite evolutionary game model

This paper constructs an evolutionary game model to explore behavioural dynamics within the four-stakeholder framework consisting of online media, netizens, short-video platforms and government regulators (figure 1). Online media, netizens and platforms aim to maximize individual benefits, while the government targets overall social welfare. Each participant's strategic choices depend on the decisions of the other three parties. Analysing the evolutionary dynamics of these stakeholders helps predict systemic changes and support timely governance interventions.

**Figure 1.**
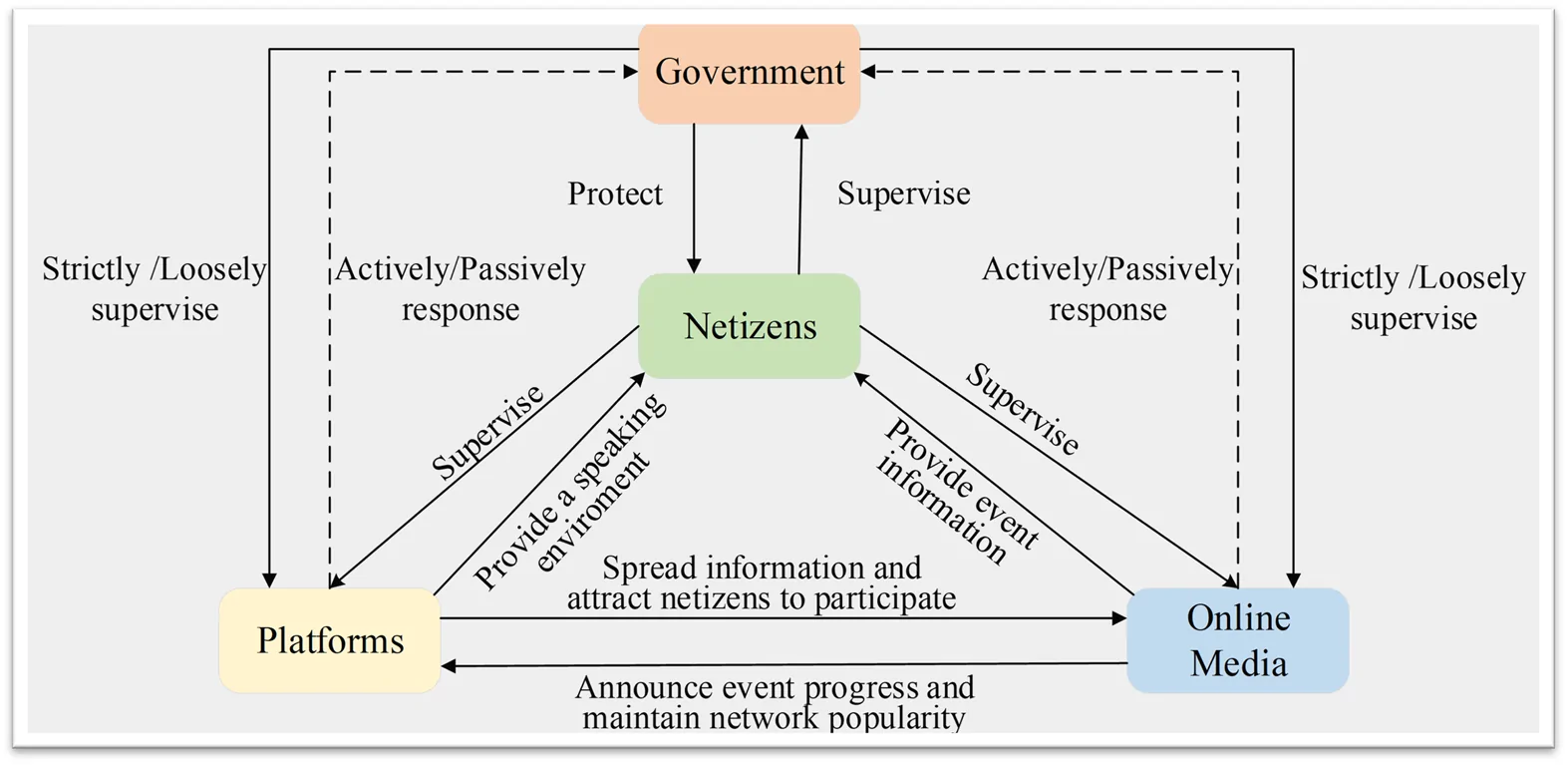
Quadripartite interaction framework among online media, netizens, short-video platforms and government regulators.

The assumptions of the proposed model are made as follows.

**Assumption 1:**

The game participants include online media, internet netizens, short-video platforms and government regulators. Let *v* denote the probability that platforms adopt active governance strategies, and 1 − *v* denote the probability of passive governance. Let *m* represent the probability that online media verifies information before release, and 1 − *m* represent the probability of releasing unchecked content. Let *g* be the probability of strict government supervision, and 1 − *g* be the probability of loose supervision. Let *n* stand for the probability that netizens rationally assess information and refuse to spread it, and 1 − *n* stand for the probability that netizens believe and disseminate unconfirmed content.

**Assumption 2:**

Short-video platforms obtain basic traffic revenue e₁₁ from user engagement (e.g. advertising income tied to views and shares). Under active governance, this revenue is sustained by a healthy digital ecosystem; under passive governance, it may still accrue when sensational unverified content drives high engagement. Platforms pay a moderation cost *c*_11_ under active governance and an ecosystem cost *c*_12_ under passive governance. Online media obtain content revenue *e*_21_ from views and shares. This revenue accrues reliably under active platform governance; under passive governance, it materializes only in the all-bad state, in which unverified content is widely believed and disseminated, while in other passive-governance states low engagement or regulatory penalties offset it. Media pay a verification cost *c*_21_ when releasing verified content and a lower production cost *c*_22_ when releasing unverified content. Netizens pay a screening cost *c*_31_ when they independently assess information. Government regulators pay *c*_41_ under strict supervision and *c*_42_ under loose supervision. The parameter a>0 is a common adjustment-speed parameter in the four replicator equations; it captures how quickly each population updates its strategy mix, is not a payoff parameter, and does not appear in table 1. The parameter b∈[0,1] denotes the probability that insiders refute false information; payoffs involving insider refutation enter as expected terms weighted by b in table 1.

**Table 1.** Quadripartite game payment matrix.

			government			
			strict supervision (*g*)		loose supervision (1 − *g*)	
strategy selection		online media	netizens question and not disseminate	netizens believe and disseminate	netizens question and not disseminate	netizens believe and disseminate
short-video platforms	active response	report after verification	e_11_ − c_11_, e_21_ − c_21_, e_32_ − c_31_, e_41_ − c_41_	e_11_ − c_11_, e_21_ − c_21_, e_31_ − c_32_, e_41_ − c_41_	e_11_ − c_11_, e_21_ − c_21_, e_32_ − c_31_, e_41_ − c_42_	e_11_ − c_11_, e_21_ − c_21_, e_31_, e_41_ − c_42_
		report immediately without verification	e_11_ − c_11_, e_21_ − c_22_ − c_24_, e_31_ − c_31_, e_42_ − c_41_	e_11_ − c_11_, e_21_ − c_22_, − c_32_, e_42_ − c_41_	e_11_ − c_11_, e_21_ − c_22_, e_32_ − c_31_, e_41_ − c_42_	e_11_ − c_11_, e_21_ − c_22_, − c_32_, e_41_ − c_42_
	negative response	report after verification	− c_12_ − bc_14_, − c_21_, e_32_ − c_31_, − c_41_	− c_12_ − c_13_, − c_21_, e_31_ − c_41_	− c_12,_ − c_21_, e_31_ − c_31_, − c_42_	− c_12_, − c_21_, e_31_, − c_42_
		report immediately without verification	− c_12_ − c_13_ − bc_14_, − c_22_ − c_23_, 0 c_13_ + c_23_ − c_41_,	− c_12_ − c_13_, − c_22_ − c_23_, 0 c_13_ + c_23_ − c_41_,	− c_12_ − bc_14_, − c_22_ − c_24_, − c_31_, − c_42_	e_11_ − c_12_ − bc_14_, e_21_ − c_22_ − bc_24_, − c_32_ + be_32_, − c_42_ − c_43_ + be_42_

**Assumption 3:**

When online media release verified content, netizens obtain benefit *e*_31_ from accessing credible information, and regulators obtain benefit *e*_41_ from a healthier information environment. If media spread false information, netizens and regulators incur losses *c*_32_ and *c*_43_, respectively. Benefits from active questioning are represented separately by *e*_32_. The full state-contingent payoffs are listed in table 1.

**Assumption 4:**

Under strict supervision, the government imposes a financial penalty *c*_23_ on media that release unverified content when the platform is passively governed. When the platform actively governs and netizens question the content, the government relies on the reputational penalty *c*_24_ rather than the financial penalty *c*_23_. The government also imposes an administrative penalty *c*_13_ on platforms with inadequate governance under strict supervision. The two platform-side terms *c*_13_ and the expected credibility loss b·*c*_14_ are distinct: *c*_13_ is the financial penalty for inadequate moderation, while b·*c*_14_ is the expected reputational loss caused by insider refutation; they may appear separately or jointly depending on the strategy profile, as enumerated in table 1.

**Assumption 5:**

Reputational loss *c*_24_ applies only to unverified content and depends on the joint strategy profile. Under active governance and loose supervision, media face no explicit *c*_24_; the cost is reflected in lower engagement. Under active governance and strict supervision, regulatory scrutiny amplifies reputational risk, so *c*_24_ applies. Under passive governance, strict supervision triggers *c*_23_ instead of *c*_24_. Under loose supervision, media face *c*_24_ when netizens question the content. If netizens do not question it, insider refutation occurs with probability b and produces the expected loss b·*c*_24_. Netizens obtain an information-literacy benefit *e*_32_ either when they critically assess content or when misinformation is refuted by insiders. Under active governance and strict supervision, active questioning directs netizens towards credible content, so *e*_31_ applies instead of *e*_32_. Regulators obtain *e*_42_ as the additional benefit generated by insider refutation, while benefits from user self-regulation are included in *e*_41_. In the all-bad state, the corresponding expected benefits are therefore b·*e*_32_ and b·*e*_42_.

Rationale of the modelling assumptions. Key governance decisions for each stakeholder are treated as dichotomous. Binary-strategy formulation renders this four-population system analytically tractable while retaining core strategic trade-offs of real-world misinformation governance. Payoff terms correspond to concrete practical governance mechanisms: *c*_11_ and *c*_12_ represent costs for active content moderation and passive/inadequate platform governance; *c*_14_ and *c*_24_ are reputation and credibility losses acting as endogenous disciplining devices, whose activation is conditional on the combined behaviours of platforms, regulators and netizens (see Assumptions 4 and 5). *c*_31_ denotes netizens’ information-discernment cost; *c*_23_ and *c*_13_are regulatory penalties targeting unverified media and poorly moderated platforms, respectively. Parameter b captures the probability and associated benefits of insider refutation and critical user assessment, as elaborated in Assumption 5. Among the five modelling assumptions, Assumptions 4 and 5 exert the strongest influence over our main stability finding: Assumption 4 defines penalty schemes for unvetted media content and insufficient platform governance, while Assumption 5 formalizes state-dependent reputation losses and insider-refutation effects. Jointly they create payoff asymmetries producing (1,0,0,1) as the unique asymptotically stable profile under benchmark parameters (§4), a result further validated by robustness checks in §5.5.

## 4. Stability analysis of system strategies and equilibrium states

### 4.1. Replicator analysis of strategies and equilibrium states

Based on the above assumptions, we establish the payoff matrix for the four-stakeholder systemic game, as shown in table 1.

According to table 1, the replication dynamic equation system can be obtained, as shown in formulas (4.1)–(4.4).

Fv=dvdt=av1−v[c12−c11+bc14+gc13+e11+m+ne11−bc14g+m(4.1)−e11gm+gn+mn−gmn 1−m1−g−c13gmn+c14bgm+n]Fm=dmdt=am1−mc22−c21−e21+bc24+gc23+ge21+nc24+ne21+ve21−bc24g−bc24n−c24gn−e21gn−bc24v−c23gv−e21gv−c24nv−e21nv+bc24gv+bc24nv+2c24gnv(4.2)+e21gnv+bc24gn−bc24gnvFg=dgdt=ag1−gmnvc42−e41−mnvc41−e41−m−1n−1v−1c42+c43−be42+nm−1v−1c13+c23−c41+c41v−1mn−c42v−1mn+mvn−1c41−e41−mvn−1c42−e41+nvm−1c41−e42−nvm−1c42−e41−m−1n−1v−1×c13+c23−c41−mn−1v−1c41+mn−1v−1c42+nm−1v−1c42(4.3)−vm−1n−1c41−e42+m−1n−1c42−e41Fn=dndt=an1−ng−1m−1v−1c31−gmvc31−e32+gmvc32−e31−gvm−1c32+gmv−1e31+mvg−1e31+gvm−1c31−e31+gmv−1c31−e32+mvg−1×c31−e32−g−1m−1v−1c32−be32+vg−1m−1c32−mg−1v−1e31(4.4)−mg−1v−1c31−e31−vg−1m−1c31−e32

The Jacobian matrix can be obtained from the above replicator-dynamic equation system:

$$\begin{array}{rl} A= & \left[\begin{array}{cccc} a\left(2v-1\right)\lambda_{1} & av\left(v-1\right)\lambda_{6} & av\left(v-1\right)\lambda_{11} & av\left(v-1\right)\lambda_{15}\\ -m\lambda_{2} & 2\left(1-2m\right)\lambda_{7} & am\left(1-m\right)\lambda_{12} & am\left(1-m\right)\lambda_{16}\\ ag\left(1-g\right)\lambda_{3} & g\lambda_{9} & a\left(1-2g\right)\lambda_{13} & ag\lambda_{17}\\ an\left(1-n\right)\lambda_{5} & -n\lambda_{10} & -n\lambda_{14} & 2a\left(1-n\right)\lambda_{18} \end{array}\right]\\ \lambda_{1}= & -\left[c_{12}-c_{11}+bc_{14}+g\left(c_{13}+e_{11}\right)+\left(m+n\right)e_{11}-bc_{14}\left(g+m\right)-e_{11}\left(gm+gn+mn-gmn\right)\right.\\ & \left.-\;c_{13}gmn+c_{14}bg\left(m+n\right)\right]\\ \lambda_{2}= & -\;a\left(1-m\right)\left[\left(e_{21}-bc_{24}\right)\left(1-g\right)\left(1-n\right)+c_{23}g-c_{24}n\left(1-2g\right)\right]\\ \lambda_{3}= & n\left(m-1\right)(c_{13}+c_{23}+e_{41}-e_{42})-\left(m-1\right)\left(n-1\right)\left(c_{13}+c_{23}+c_{42}+c_{43}-e_{42}-be_{42}\right)\\ \lambda_{5}= & gmc_{32}+g\left(m-1\right)\left(c_{31}-e_{31}-c_{32}\right)+m\left(g-1\right)\left(e_{31}-e_{32}\right)+\left(g-1\right)\left(m-1\right)\left(1+b\right)e_{32}\\ \lambda_{6}= & -[e_{11}\left(1-g\right)\left(1-n\right)-bc_{14}\left(1-g\right)-c_{13}gn\\ \lambda_{7}= & c_{22}-c_{21}-e_{21}+bc_{24}+gc_{23}+ge_{21}+nc_{24}+ne_{21}+ve_{21}-bc_{24}g-bc_{24}n-c_{24}gn-e_{21}gn-bc_{24}v-c_{23}gv\\ & -\;e_{21}gv-c_{24}nv-e_{21}nv+bc_{24}gv+bc_{24}nv+2c_{24}gnv+e_{21}gnv+bc_{24}gn-bc_{24}gnv\\ \lambda_{9}= & a\left(1-g\right)\left[nv\left(e_{41}-e_{42})+n\left(1-v\right)\left(c_{13}+c_{23}\right)+v\left(n-1\right)\left(e_{42}-c_{42}\right)-\left(n-1\right)(v-1\right)\right.\\ & \times \left.\left(c_{13}+c_{23}+c_{43}-be_{42}\right)+\left(n-1\right)\left(c_{42}-e_{41}\right)\right]\\ \lambda_{10}= & -\;a\left(1-n\right)\left[gv\left(e_{32}-2e_{31}\right)+g\left(v-1\right)\left(e_{31}+c_{31}-e_{32}\right)+v\left(g-1\right)\left(e_{31}+c_{32}\right)\right.\\ & \left.+\left(g-1\right)\left(v-1\right)\left(be_{32}-c_{32}\right)\right]\\ \lambda_{11}= & -[c_{13}\left(1-mn\right)+e_{11}\left(1-m\right)\left(1-n\right)-bc_{14}+c_{14}b\left(m+n\right)\\ \lambda_{12}= & \left(1-v\right)[c_{23}+\left(e_{21}-bc_{24}\right)\left(1-n\right)]-c_{24}n\left(1-2v\right)\\ \lambda_{13}= & mnv\left(c_{42}-e_{41}\right)-mnv\left(c_{41}-e_{41}\right)-\left(m-1\right)\left(n-1\right)\left(v-1\right)\left(c_{42}+c_{43}-be_{42}\right)+n\left(m-1\right)\left(v-1\right)\\ & \times \left(c_{13}+c_{23}-c_{41}\right)+c_{41}\left(v-1\right)mn-c_{42}\left(v-1\right)mn+mv\left(n-1\right)\left(c_{41}-e_{41}\right)-mv\left(n-1\right)\left(c_{42}-e_{41}\right)\\ & +\;nv\left(m-1\right)\left(c_{41}-e_{42}\right)-nv\left(m-1\right)\left(c_{42}-e_{41}\right)-\left(m-1\right)\left(n-1\right)\left(v-1\right)\left(c_{13}+c_{23}-c_{41}\right)-m\left(n-1\right)\\ & \times \left(v-1\right)c_{41}+m\left(n-1\right)\left(v-1\right)c_{42}+n\left(m-1\right)\left(v-1\right)c_{42}-v\left(m-1\right)\left(n-1\right)\left(c_{41}-e_{42}\right)\\ & +\left(m-1\right)\left(n-1\right)\left(c_{42}-e_{41}\right)\\ \lambda_{14}= & -\;a\left(1-n\right)\left[mvc_{32}+m\left(v-1\right)\left(e_{31}-e_{32}\right)+v\left(m-1\right)\left(e_{32}-e_{31}\right)+\left(m-1\right)\left(v-1\right)\left(c_{31}-c_{32}+be_{32}\right)\right]\\ \lambda_{15}= & -\left[e_{11}\left(1-g\right)\left(1-m\right)-c_{13}gm+c_{14}bg\right]\\ \lambda_{16}= & \left(e_{21}-bc_{24}\right)\left(1-g\right)\left(1-v\right)+c_{24}\left(1-g-v+2gv\right)\\ \lambda_{17}= & \left(1-g\right)\left(m-1\right)\left[v\left(e_{41}-c_{42}\right)+\left(v-1\right)\left(be_{42}-c_{43}\right)+\left(c_{42}-e_{41}\right)\right]\\ \lambda_{18}= & \left(g-1\right)\left(m-1\right)\left(v-1\right)c_{31}-gmv\left(c_{31}-e_{32}\right)+gmv\left(c_{32}-e_{31}\right)-gv\left(m-1\right)c_{32}+gm\left(v-1\right)e_{31}\\ & +\;mv\left(g-1\right)e_{31}+gv\left(m-1\right)\left(c_{31}-e_{31}\right)+gm\left(v-1\right)\left(c_{31}-e_{32}\right)+mv\left(g-1\right)\left(c_{31}-e_{32}\right)-\left(g-1\right)\\ & \times \left(m-1\right)\left(v-1\right)\left(c_{32}-be_{32}\right)+v\left(g-1\right)\left(m-1\right)c_{32}-m\left(g-1\right)\left(v-1\right)e_{31}-m\left(g-1\right)\left(v-1\right)\\ & \times \left(c_{31}-e_{31}\right)-v\left(g-1\right)\left(m-1\right)\left(c_{31}-e_{32}\right) \end{array}$$

Using the payoff matrix in table 1, equations (4.1)–(4.4) describe the replicator dynamics of the four populations. Solving these equations yields 16 pure-strategy equilibria and the corresponding mixed-strategy equilibria. Throughout this section, the state vector is ordered as (v, m, g, n).

Strict Nash equilibria in this asymmetric multi-population game are pure-strategy profiles. Since a strict Nash equilibrium is locally asymptotically stable under replicator dynamics, we first evaluate the Jacobian matrix at the 16 vertices of the state space [0,1]⁴. A vertex is locally asymptotically stable if all four eigenvalues are negative. Positive eigenvalues indicate instability, whereas zero eigenvalues produce non-hyperbolic cases that require separate numerical treatment. Tables 2 and 3 summarize the results for the passive and active platform-governance states. Interior equilibria are examined separately in §4.3.

**Table 2.** Stability of equilibria under passive platform governance (*v* = 0).

equilibrium points	Eigenvalue				stability
	λ_1_	λ_2_	λ_3_	λ_4_	
(0,0,0,0)	− 0.63	− 16.17	17.15	0.63	unstable
(0,0,0,1)	22.47	7.70	4.20	− 0.63	unstable
(0,0,1,0)	25.20	− 2.10	− 17.15	0	non-hyperbolic (indicative)
(0,0,1,1)	32.97	− 2.10	− 4.20	0	non-hyperbolic (indicative)
(0,1,0,0)	14.70	16.17	− 12.60	− 10.50	unstable
(0,1,0,1)	14.70	− 7.70	− 12.60	10.50	unstable
(0,1,1,0)	25.20	2.10	12.60	− 9.80	unstable
(0,1,1,1)	22.47	2.10	12.60	9.80	Unstable(source)

**Table 3.** Stability of equilibria under active platform governance (*v* = 1).

equilibrium points	Eigenvalue				stability
	λ_1_	λ_2_	λ_3_	λ_4_	
(1,0,0,0)	0.63	− 8.40	− 11.20	7.00	unstable
(1,0,0,1)	− 22.47	− 8.40	− 11.20	− 7.00	Stable(ESS)
(1,0,1,0)	− 25.20	− 8.40	11.20	6.30	unstable
(1,0,1,1)	− 32.97	7.70	11.20	− 6.30	unstable
(1,1,0,0)	− 14.70	8.40	− 12.60	− 9.80	unstable
(1,1,0,1)	− 14.70	8.40	− 12.60	9.80	unstable
(1,1,1,0)	− 25.20	8.40	12.60	2.80	unstable
(1,1,1,1)	− 22.47	− 7.70	12.60	− 2.80	unstable

*Notes:* The two equilibria (0,0,1,0) and (0,0,1,1) in table 2 are non-hyperbolic with a zero eigenvalue along the netizens direction; their classification is indicative only. Numerical verification (100 initial conditions within ±0.05 of each point) provides numerical evidence that neither equilibrium is attracting, and the unique asymptotically stable state of the system is the ESS (1,0,0,1).

### 4.2. Stability of pure-strategy equilibria

Table 2 shows that no corner equilibrium on the v = 0 face is asymptotically stable. Apart from (0,1,1,1), which is an unstable source, the remaining seven corners are unstable saddle points or non-hyperbolic states. The two non-hyperbolic equilibria (0,0,1,0) and (0,0,1,1) have a positive eigenvalue in the platform direction. Numerical tests from 100 initial conditions within ±0.05 of each point provide evidence that neither equilibrium is attracting. Under the benchmark parameters, passive platform governance therefore does not support a stable corner equilibrium.

When v = 1, the Jacobian analysis identifies (1,0,0,1) as the only asymptotically stable corner. Its four eigenvalues, [−22.47, −8.40, −11.20, −7.00], are all negative. At this profile, platforms adopt active governance, online media report without verification, government regulators exercise loose supervision and netizens assess information rationally and avoid indiscriminate sharing. All other corners on the v = 1 face are unstable. For example, (1,0,1,0) has two positive eigenvalues and is a saddle. Under the benchmark parameter set, the only stable corner is therefore platform-led governance supported by rational netizens and limited direct supervision.

### 4.3. Interior equilibria

The absence of a stable interior equilibrium reported here is a model-specific result, not a general property of asymmetric evolutionary games. In symmetric two-strategy games such as the Chicken (Snowdrift) game, the two strategy frequencies belong to the same population and satisfy the simplex constraint *y* = 1 − *x*. This constraint reduces the dynamics to a single dimension, and under appropriate dilemma conditions an interior equilibrium can be locally attracting through the one-dimensional sign-reversal mechanism. In the present model, the four populations evolve independently on [0, 1]^4^, and no analogous simplex constraint links *v*, *m*, *g* and *n.* An interior equilibrium must therefore satisfy all four replicator equations simultaneously, and its local stability must be assessed from the full four-dimensional Jacobian. Because the four populations do not share a single simplex, the standard scalar dilemma-strength measure developed for symmetric two-player games cannot be directly applied here; the relevant stability condition is the sign pattern of the four-dimensional Jacobian rather than a one-dimensional dilemma-strength scalar. The one-dimensional sign-reversal mechanism that stabilizes interior equilibria in symmetric games therefore cannot operate directly in the present four-population setting. The cited works are used as a comparative theoretical background for understanding why symmetric and asymmetric systems differ in this respect, not as a direct derivation of the stability results reported below [31–35].

While the absence of a simplex constraint does not preclude interior stability in every asymmetric game, no asymptotically stable interior equilibrium arises in the present model. Under the benchmark parameters, our numerical search did not identify an interior equilibrium; additionally, all 1296 grid-based initial conditions converged to (1,0,0,1). To probe robustness, we sample 200 perturbed parameter sets. In 43 of these, an interior equilibrium appears, but 32 are saddle points and 11 are non-hyperbolic; none is asymptotically stable. Interior equilibria were located by solving the four replicator equations simultaneously using MATLAB's fsolve, with multiple initial guesses sampled uniformly across the interior of [0,1]⁴; each solution was then verified by checking that the residuals of all four equations were below 10⁻⁸, and its stability was assessed from the eigenvalues of the four-dimensional Jacobian. Within the tested parameter range, (1,0,0,1) therefore remains the unique asymptotically stable equilibrium. The absence of a stable interior equilibrium is thus specific to the payoff structure and parameterization adopted here, rather than a generic property of asymmetric evolutionary games.

The stability of (1,0,0,1) hinges on the payoff asymmetry encoded in Assumptions 4 and 5. Financial and administrative penalties bear on unverified content and inadequately moderated platforms; contingent reputational loss and insider refutation then punish the deviations that survive those penalties. Together, these terms align the four populations' incentives on (1,0,0,1). How sensitive this outcome is to the underlying cost and benefit parameters is examined next.

## 5. Numerical simulation and sensitivity analysis

We simulate the replicator dynamics in MATLAB to examine how changes in key costs and benefits shape convergence and equilibrium selection. Table 4 reports the baseline parameters, whose relative magnitudes follow prior governance and evolutionary-game studies; the analysis below focuses on qualitative dynamics and comparative statics rather than quantitative prediction.

**Table 4.** Relevant parameters and their initial values.

parameter	initial value	parameter	initial value	parameter	initial value
*e*_11_	33	*c*_31_	15	*c*_23_	9
*c*_11_	15	*c*_41_	24	*c*_14_	37
*c*_12_	3	*c*_42_	6	*c*_24_	23
*e*_21_	18	*c*_32_	18	*e*_32_	7
*c*_21_	22	*c*_43_	30	*e*_42_	15
*c*_22_	10	*c*_13_	15	*a*	0.7
*e*_31_	6	*e*_41_	13	*b*	0.3

*Notes:* Baseline values are illustrative rather than empirically calibrated, and preserve the intended relative ordering of costs and benefits: platform revenue and credibility/reputation losses are largest (order 20–40), regulatory penalties and user screening costs are intermediate (order 10–20), and production and information benefits are smallest (order 3–10). The adjustment-speed parameter *a* = 0.7 and insider-refutation probability *b* = 0.3 are set below unity (*b*∈ [0,1]). All qualitative results are robust to ±30% perturbations (§5.5).

### 5.1. Effect of platform traffic revenue *e*_11_

The system converges to (1,0,0,1) across all three values of *e*_11_. Higher *e*_11_ accelerates the rise of *v* and *n* towards one, and drives m to zero faster. The clearest contrast appears in *g*: at *e*_11_ = 5, *g* initially rises before decaying, whereas at *e*_11_ = 20 and 40, it declines monotonically. This pattern indicates that platform governance and user rationality substitute for direct regulatory pressure as platform traffic revenue grows.

### 5.2. Effect of the insider-refutation probability *b*

All three values of *b* lead to (1,0,0,1). As *b* rises, active platform governance and rational user assessment emerge faster, and strict government supervision declines sooner. Media verification also falls more quickly, since active platform governance and rational netizens already constrain misinformation in the model. Timely insider refutation therefore substitutes for prolonged strict supervision.

### 5.3. Effect of credibility and reputation losses *c*_14_ and *c*_24_

At larger (*c*_14_,*c*_24_), the system reaches (1,0,0,1) faster: v and *n* rise more quickly, while *m* and *g* decline. The two parameters operate through different channels. Larger *c*_14_ strengthens the platform's incentive to protect its credibility, accelerating *v*. Larger *c*_24_, however, does not raise media verification; once active platform governance and rational netizens already reduce misinformation, additional media verification has little marginal value.

### 5.4. Effect of government supervision

To isolate the effect of government supervision, we fix g at two levels *g* = 0 and *g* = 0.8, and launch 100 trajectories from uniformly random initial conditions in (*v*, *m*, *n*) ∈ [0,1]³. Figure 2 reports the trajectories under each fixed level.

**Figure 2.**
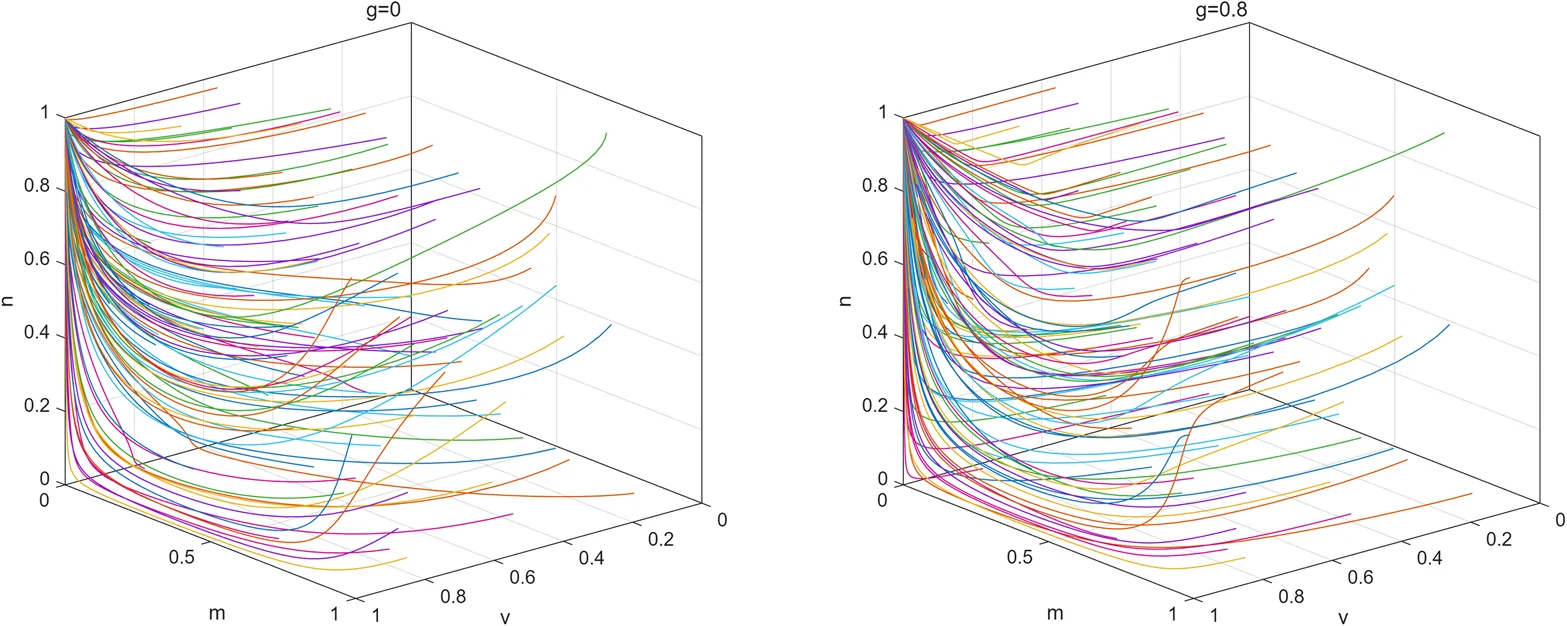
Evolutionary trajectories of the four stakeholder strategies under fixed government supervision *g* = 0 and *g* = 0.8, with 100 trajectories launched from uniformly random initial conditions in [0,1]³ for (*v*, *m*, *n*), and all other parameters as in table 4.

Under the benchmark payoff structure, trajectories from the sampled initial conditions converge towards *v* = 1, *m* = 0 and *n* = 1 in both panels. The fixed level of government supervision changes only the transient path: *g* = 0.8 accelerates the decline of m and the rise of *v* relative to *g* = 0, but neither the long-run equilibrium nor its stability is affected. Within the model, strict supervision functions as a complementary transitional mechanism rather than a permanent requirement. During the early phase, it accelerates the emergence of active platform governance and rational user assessment; once these endogenous mechanisms are established, the simulations indicate that continued strict supervision is no longer necessary for maintaining the benchmark equilibrium. We emphasize that this is a model-based finding, not an empirical prescription for real-world regulatory timing. The long-run target is therefore not perpetual strict supervision, but a self-sustaining configuration in which active platform governance and user critical assessment internalize the governance problem, with the regulator reverting to a lighter-touch posture. Across all simulations in figures 2–5, the strategy frequencies converge monotonically to the benchmark equilibrium without sustained overshoot or oscillatory behaviour.

**Figure 3.**
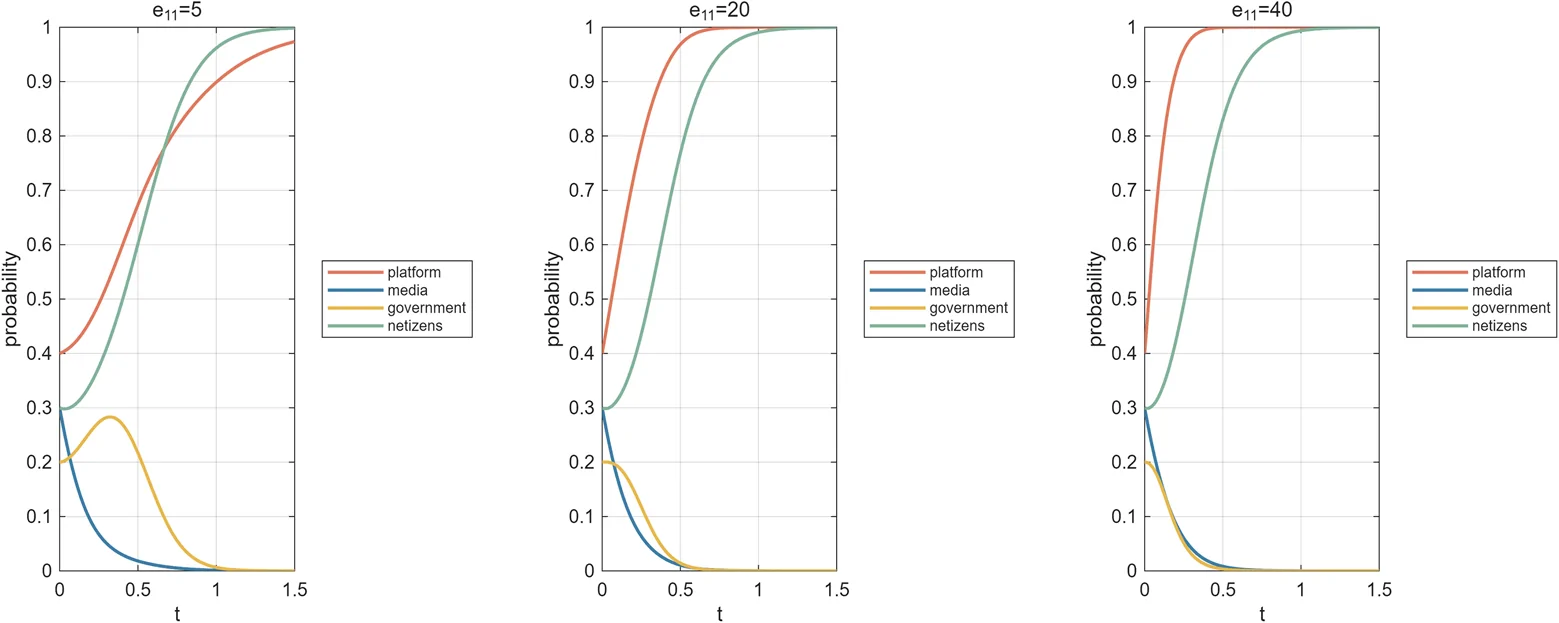
Evolutionary trajectories of the four stakeholder strategies—platform active governance (*v*), media verification (*m*), strict government supervision (*g*), and netizens' rational assessment (*n*)—platform traffic revenue *e*_11_= 5, 20 and 40, with all other parameters as in table 4 and initial condition (*v*, *m*, *g*, *n*) = (0.4, 0.3, 0.2, 0.3).

**Figure 4.**
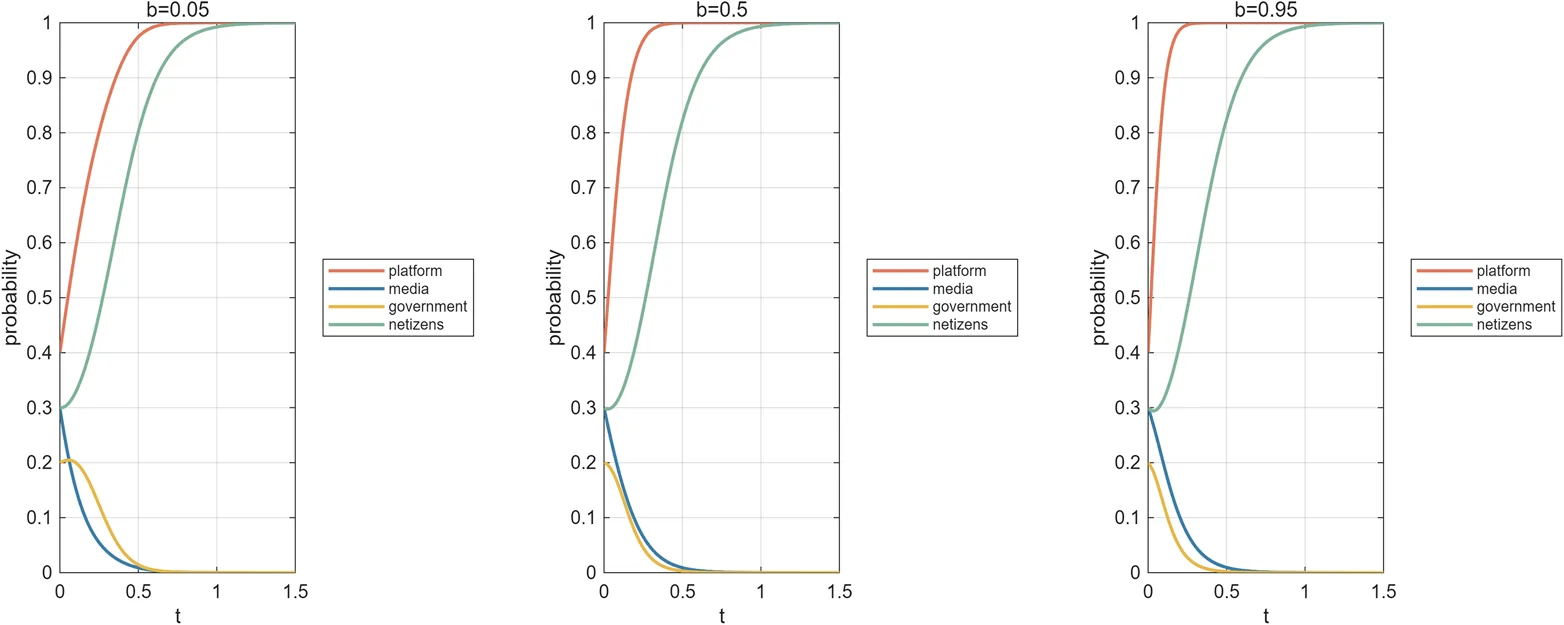
Evolutionary trajectories of the four stakeholder strategies—platform active governance (*v*), media verification (*m*), strict government supervision (*g*) and netizens' rational assessment (*n*)—over time for the insider-refutation probability *b* = 0.05, 0.5 and 0.95, with all other parameters as in table 4 and initial condition (*v*, *m*, *g*, *n*) = (0.4, 0.3, 0.2, 0.3).

**Figure 5.**
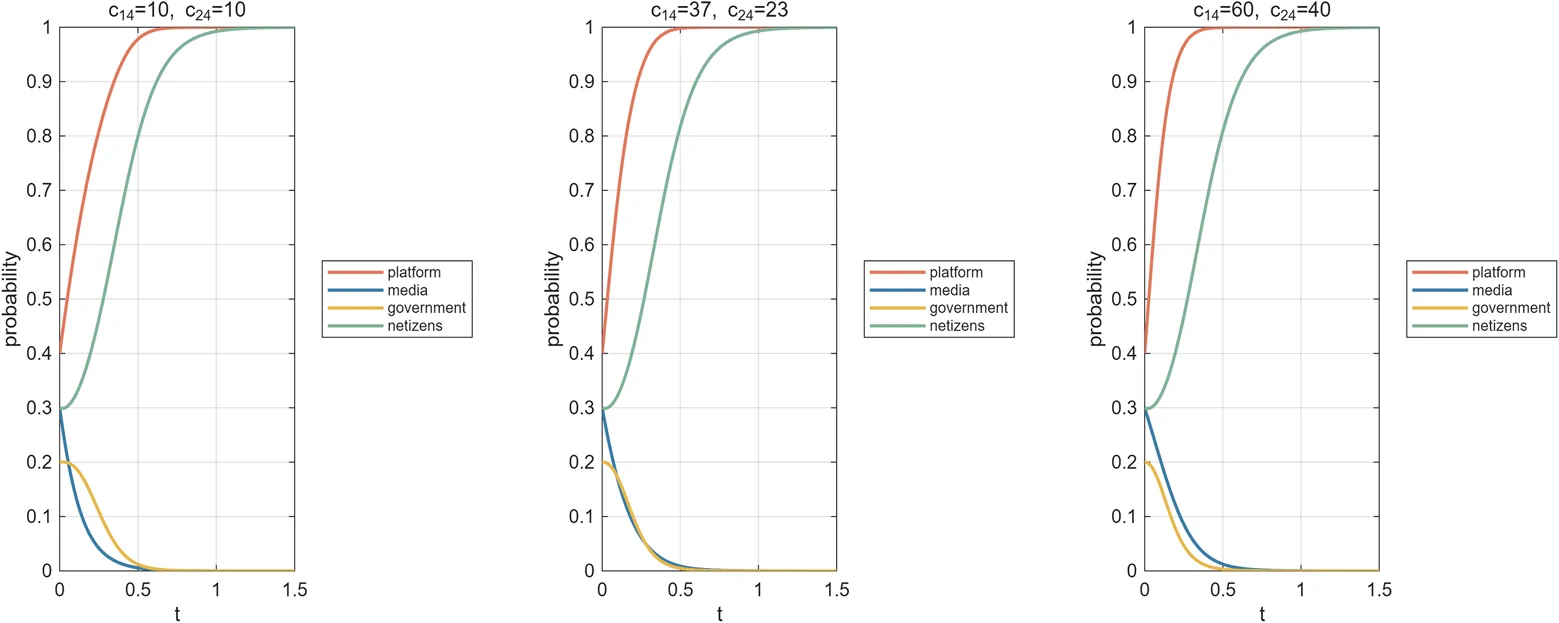
Presents the trajectories for (c_14_,c_24_) = (10,10), (37,23), (60,40). Higher values of both parameters accelerate convergence to (1,0,0,1). The platform moves towards active governance and netizens towards rational assessment more quickly, while media verification and strict government supervision decline faster.

### 5.5. Robustness check

To examine whether the ESS conclusion is sensitive to parameter choice, we simultaneously perturb all 21 baseline parameters, each by an independently drawn multiplicative factor from a uniform distribution on [0.7, 1.3] (i.e. up to ±30%), and re-solve the Jacobian eigenvalues at all 16 pure-strategy corner equilibria across 200 parameter sets. The probability parameter *b* is truncated to [0,1] after perturbation. All draws use a fixed random seed (seed = 7) for reproducibility; the full robustness-check script is available in the Zenodo repository [36]. In 197 of the 200 perturbed parameter sets (98.5%), (1,0,0,1) remains the unique asymptotically stable equilibrium with all four eigenvalues strictly negative. In the remaining three sets, two converge to (1,0,0,0) as the stable corner and one has no asymptotically stable corner; no parameter set yields multiple stable corners or a stable interior equilibrium. Inspection of the three anomalous sets reveals a common pattern: the user's screening cost is perturbed upward by 26%–29%, while the user's misinformation loss and information-literacy benefit are perturbed downward by 17%–30% and 17%–24%, respectively. Under this extreme combination, the net benefit of rational information assessment becomes negative, so the user-irrational corner (1,0,0,0) replaces (1,0,0,1) as the stable profile. This boundary case occurs only when all three user-side parameters simultaneously approach their ±30% perturbation limits in the unfavourable direction; it does not arise under more moderate perturbations. Thus, within the tested range, (1,0,0,1) is the overwhelmingly dominant stable profile, and its stability is robust except in an extreme corner of the parameter space in which rational user assessment is no longer individually beneficial.

The MATLAB scripts implementing this robustness analysis are archived in Zenodo [36].

## 6. Conclusion

### 6.1. Main findings

This study examines how misinformation governance evolves in short-video socio-technical systems with four stakeholder populations. Under the benchmark parameters, equilibrium analysis identifies (1,0,0,1) as the unique asymptotically stable profile. At this profile, platforms govern actively, online media report without verification, government regulators exercise loose supervision, and netizens assess information rationally. We refer to this outcome as the platform-led governance equilibrium. The simulations indicate that active platform governance and user rationality are the main sources of stability in the model. Government supervision primarily changes the incentive structure and the transient evolutionary path. It can accelerate convergence while strict measures are in place, but sustained strict supervision is not required once platform governance and user rationality are established. Government supervision therefore acts as a complementary mechanism rather than a substitute for platform governance or user information literacy.

### 6.2. Theoretical contributions

The replicator dynamics used here are well established; the contribution lies in applying them to a setting in which short-video platforms make strategic decisions that interact with media, netizens and regulators. Three contributions follow.

First, it models four stakeholder populations and treats the short-video platform as a strategic actor rather than a passive channel. This extends earlier two- and three-party frameworks, in which platform responses were typically left implicit, and captures the interdependence among online media, netizens, platforms and regulators under bounded rationality. Second, the analysis shows how endogenous mechanisms of user rationality and reputation loss interact with government supervision and penalties to drive convergence. Across the tested parameter ranges, active platform governance and user rationality are the main drivers of convergence, while government supervision mainly shapes the incentive environment and the transition path. Third, the framework is transferable to other multi-stakeholder governance settings in socio-technical systems. It identifies the conditions under which a self-organizing governance equilibrium can emerge, and the same structure can be adapted to analogous digital-governance problems.

### 6.3. Policy and system implications

Because the model is theoretical and is not calibrated to platform data, the implications below should be read as model-based propositions rather than direct policy evidence. Two boundaries are worth stating upfront. First, the model explicitly represents platform governance, regulatory supervision, insider refutation and user critical assessment; it does not separately parameterize algorithmic recommendation or formal user-education programmes, so these cannot be evaluated as independent policy instruments here. Second, platform governance in the model is narrowly defined as the active decision to verify and remove unverified content, and should not be conflated with recommendation design or third-party fact-checking. Subject to these boundaries, the results point to three priorities, discussed in turn below.

Platform governance is the most direct lever for stability in the model. Policy attention should therefore focus on the costs and incentives that shape platform moderation decisions; penalties on inadequate moderation and unverified media content can reinforce the shift towards the platform-led equilibrium. User information literacy is a second internal stabilizing mechanism. Measures that improve users’ ability to assess and resist misinformation reduce reliance on strict supervision and accelerate convergence to the stable profile. Government supervision, by contrast, is best viewed as a transitional and complementary mechanism. Penalties and monitoring can strengthen incentives for active platform governance, but the model does not indicate that continuous strict supervision is necessary; supervision is important for steering the system towards the stable profile, while its long-run role diminishes as platform governance and user rationality strengthen. In combination, platform governance and user information literacy form the main internal stabilizing mechanism, with government enforcement providing complementary support.

### 6.4. Limitations and future research

The interpretation of these results is subject to three main limitations. First, the analysis is theoretical and relies on illustrative parameter values drawn from existing governance and evolutionary-game literature, rather than operational platform data or statistically estimated real-world values. Second, the binary strategy set omits mixed, gradual or delayed strategic responses. Third, the model does not include algorithmic recommendation, cross-platform diffusion or individual emotional factors. These omissions may affect both convergence speed and equilibrium selection in real systems.

Future research could estimate parameters using platform behavioural data, allow mixed or graded strategies and incorporate algorithmic and emotional mechanisms. These extensions would test whether the platform-led equilibrium persists under more realistic information environments and whether additional stability conditions emerge.

## Supplementary Material



## Data Availability

All MATLAB simulation scripts, payoff-matrix documents, parameter tables, equilibrium-solving codes and trajectory-plotting programs used to generate the numerical results are archived in Zenodo [36]. All code and supporting files in this Zenodo archive are released under the CC-BY 4.0 licence. Supplementary material is available online [37].

## Declaration of AI use

AI-assisted tools were only used for Chinese–English translation, grammar correction and academic English polishing. None of the core theoretical modelling, evolutionary game equations, MATLAB simulation codes, equilibrium analysis, data interpretation or research conclusions were generated by AI. All AI-revised text was manually reviewed and modified by authors to preserve academic originality and logical rigour. The proportion of AI-generated content within the full manuscript is less than 5%.
