# Supplementary material for: Evolutionary dynamics of a four-stakeholder ecosystem for misinformation governance on short-video platforms
Source: R Soc Open Sci. 2026 Oct 28;13(10):261361. doi: 10.1098/rsos.261361 (PMC13714372; doi:10.1098/rsos.261361)
Supplement: Supplementary file 1 [file rsos.261361.zip › rsos.261361/rsos261361_si_001.docx]

**Evolutionary dynamics of a four-stakeholder ecosystem for misinformation governance on short-video platforms**

Xiaoyu Song^1^ Wenjie Song^2*^

1. School of Economics and Management, Yanshan University, Qinhuangdao 066000, China
2. School of Public Administration, Yanshan University, Qinhuangdao 066000, China

**Corresponding Author:** Wenjie Song

School of Public Administration, Yanshan University, Qinhuangdao 066000, China

**E-mail:** vivianY0302@163.com

**Journal:** Royal Society Open Science

**Article DOI:** 10.1098/rsos.261361 [RSOS-261361]

**Contents:**

1. MATLAB scripts used for all numerical simulations and analyses

2. Figures 1–5

**Description:**

This supplementary material contains the complete MATLAB code used to generate all numerical results in the main text, together with Figures1–5. The scripts reproduce the equilibrium and stability calculations, the evolutionary trajectories shown in Figures 2–5, and the robustness analysis reported in Section 4.5 of the main text.

**Data and code availability:**

The MATLAB scripts used in this study are also deposited in the Zenodo repository: [https://doi.org/10.5281/zenodo.22749192]
